# Promoting health in the digital environment: health policy experts’ responses to on-demand delivery in Aotearoa New Zealand

**DOI:** 10.1093/heapro/daad091

**Published:** 2023-08-23

**Authors:** Christina McKerchar, Susan Bidwell, Angela Curl, Tessa Pocock, Matt Cowie, Hannah Miles, Rose Crossin

**Affiliations:** Department of Population Health, University of Otago Christchurch, Christchurch, New Zealand; Department of Population Health, University of Otago Christchurch, Christchurch, New Zealand; Department of Population Health, University of Otago Christchurch, Christchurch, New Zealand; Department of Population Health, University of Otago Christchurch, Christchurch, New Zealand; School of Nursing, Faculty of Medical and Health Sciences, The University of Auckland, Auckland, New Zealand; University of Otago Medical School, Dunedin, New Zealand; University of Otago Medical School, Dunedin, New Zealand; Department of Population Health, University of Otago Christchurch, Christchurch, New Zealand

**Keywords:** digital environment, alcohol, food, smoking, health promoting environments

## Abstract

Services offering on-demand delivery of unhealthy commodities, such as fast food, alcohol and smoking/vaping products have proliferated in recent years. It is well known that the built environment can be health promoting or harmful to health, but there has been less consideration of the digital environment. Increased availability and accessibility of these commodities may be associated with increased consumption, with harmful public health implications. Policy regulating the supply of these commodities was developed before the introduction of on-demand services and has not kept pace with the digital environment. This paper reports on semi-structured interviews with health policy experts on the health harms of the uptake in on-demand delivery of food, alcohol and smoking/vaping products, along with their views on policies that might mitigate these harms. We interviewed 14 policy experts from central and local government agencies and ministries, health authorities, non-Government Organisations (NGOs) and university research positions in Aotearoa New Zealand using a purposive sampling strategy. Participants concerns over the health harms from on-demand services encompassed three broad themes—the expansion of access to and availability of unhealthy commodities, the inadequacy of existing restrictions and regulations in the digital environment and the expansion of personalized marketing and promotional platforms for unhealthy commodities. Health policy experts’ proposals to mitigate harms included: limiting access and availability, updating regulations and boosting enforcement and limiting promotion and marketing. Collectively, these findings and proposals can inform future research and public health policy decisions to address harms posed by on-demand delivery of unhealthy commodities.

**Contribution to Health Promotion**

- Goods and services that people have access to in their neighbourhood can promote or harm health.
- On-demand services connect customers with products like meals and alcohol, with delivery under 2 h, directly to the door. Customers can access a broader range of items than traditionally available within their local neighbourhood.
- Our research investigates potential impacts public health and the importance of considering the digital environment from a health promotion perspective.
- Health policy experts in Aotearoa New Zealand told us about their concerns for public health and gave suggestions for changes to regulations that might help reduce harm.

## INTRODUCTION

Poor diet, over-consumption of alcohol, smoking and obesity are major contributors to cardiovascular diseases, diabetes, chronic respiratory diseases and some cancers worldwide (Griswold *et al.*, 2018; Afshin *et al.*, 2019; World Health Organisation, 2021). Globally, there are health inequities in relation to ethnicity and socio-economic deprivation, and in New Zealand there are persistent health inequities between Māori and non-Māori (Baker *et al.*, 2019). A key factor in modifying the risk of these noncommunicable diseases is reducing the consumption of unhealthy commodities: unhealthy foods high in added sugar, saturated fat and sodium, excess alcohol, smoking and vaping products. Efforts to this end have long been a primary focus of health promotion policies and interventions [e.g. (Sacks *et al.*, 2009; Yu *et al.*, 2018; WHO, 2019)]. Moreover, there is increasing recognition that policies that alter the environment result in better health outcomes than those that focus on the individual, and are likely to be more cost-effective and reduce inequities over the long term (Gortmaker *et al.*, 2015; Moran *et al.*, 2020). Such policies include restrictions on access to and availability of fast food, alcohol and smoking/vaping products, restrictions on sponsorship and advertising, and proactive approaches that incentivize healthier choices (Kauppila *et al.*, 2019; Katainen *et al.*, 2020; Scobie *et al.*, 2022).

Online services (via websites and/or apps) that enable rapid on-demand delivery of food, alcohol and smoking/vaping products (usually in less than 2 h) (Duthie *et al.*, 2023) are expanding both globally (Pinho *et al.*, 2020) and in Aotearoa New Zealand (Miles *et al.*, 2022). Such on-demand services typically coordinate between consumers, multiple outlets and third-party delivery services. Consumers enjoy the efficiency and convenience of being able to order from a wide range of sellers through a single on-demand service, and companies have moved quickly to meet demand (Zhao *et al.*, 2020; Galanakis *et al.*, 2021). Across Aotearoa New Zealand, Uber Eats operates on-demand food delivery from multiple sellers in 55 cities and towns (Uber Eats, 2022). Rapid alcohol delivery services have also proliferated, either as a standalone service or provided through on-demand meal delivery services (Huckle *et al.*, 2020; Miles *et al.*, 2022), while delivery of vaping products has also entered the on-demand space (Miles *et al.*, 2022). Although on-demand delivery dates back more than a decade, the stay-home orders during the COVID-19 pandemic generated an explosion in consumer uptake (Gaiha *et al.*, 2020; Huckle *et al.*, 2020; Fitzgerald *et al.*, 2022; Grossman *et al.*, 2022) that has persisted and continues to increase (Casswell *et al.*, 2021).

Many studies globally have highlighted the health harms and inequities inherent in the increase in access and availability of unhealthy commodities. Analysis of the food offered through on-demand delivery services has consistently found it to be unhealthy, with the most popular items being ‘discretionary foods’ that are high in energy, fat, sodium and sugar but low in nutritional value (Horta *et al.*, 2020; Partridge *et al.*, 2020; Poelman *et al.*, 2020; Halloran *et al.*, 2021; Wang *et al.*, 2021; Keeble *et al.*, 2022; Norriss *et al.*, 2022). A recent report by the World Health Organisation (WHO, 2021) termed on-demand services with a high concentration of unhealthy food options as ‘digital food swamps’. The declaration of alcohol as an essential product during the COVID-19 pandemic in Aotearoa New Zealand and other countries has normalized and entrenched online home deliveries, which may have inadvertently encouraged increased alcohol consumption (Colbert *et al.*, 2020; Huckle *et al.*, 2020; Matthay and Schmidt, 2021; Fitzgerald *et al.*, 2022). Smoking and vaping products are also available via on-demand delivery, potentially increasing the likelihood of youth uptake (Gurram *et al.*, 2019; Meacham *et al.*, 2020; Miles *et al.*, 2022). The uptake of on-demand services for ordering unhealthy commodities (food, alcohol, smoking/vaping products) has been viewed as creating unintended consequences for vulnerable sectors of society (Louie *et al.*, 2022; Parsons and Barling, 2022), potentially compounding inequity for people living in more deprived areas who are already likely to have negative health outcomes (Hay *et al.*, 2009; Vandevijvere *et al.*, 2018; Wiki *et al.*, 2019; Keeble *et al.*, 2021). In Aotearoa New Zealand, Māori are more likely than non-Māori to live in deprived areas and are therefore potentially at greater risk of harm resulting from the expanding digital environment.

In parallel with the uptake of on-demand delivery services there has been a proliferation of personalized marketing and promotion of unhealthy commodities (Williams and Schmidt, 2014; Norman *et al.*, 2016; Gurram *et al.*, 2019; Montgomery *et al.*, 2019; Kelly *et al.*, 2021). A WHO briefing paper found that digital marketing of alcohol increasingly uses covert methods, identifying and pursuing individuals who are most vulnerable to developing alcohol use disorders, and using techniques of paid marketing combined with strategies that encourage consumers to internalize brands and promote them on their social media platforms (Casswell *et al.*, 2021). Marketing to children through social media has been described as infringing children’s rights, failing to protect their best interests, behavioural targeting, manipulation and violating their right to privacy from the sale of their personal data (Tatlow-Golden and Garde, 2020).

Efforts to coordinate policy in relation to on-demand delivery services and address the gaps in regulations that were designed for the built environment have been slow (Montgomery *et al.*, 2019; Tatlow-Golden and Garde, 2020; Jankhotkaew *et al.*, 2022; Ling *et al.*, 2022). The WHO has termed this lack of regulation a ‘blind spot’ (Carlin *et al.*, 2021), concluding that in ‘almost all cases current policy and legal frameworks do not apply’ to them [(World Health Organisation, 2021), p. vi].

This statement also applies to regulatory regimes in Aotearoa New Zealand. Food sales and premises are regulated for safety and suitability by the Food Act (New Zealand Government, 2014) and administered by the Ministry of Business, Innovation and Employment (MBIE). Regulation is designed to keep food safe to eat and free from contamination, and currently has no mechanism for considering the nutritional quality of the food sold. The sale and supply of alcohol and smoking and vaping products is regulated by government legislation (New Zealand Government, 2012, 2020b). Monitoring of compliance with the law (known as ‘controlled purchases’) for smoking and vaping products is done by Ministry of Health staff stationed in regional health offices and for alcohol by authorized staff from local authorities, however, monitoring is not carried out for online sales. A feature of Aotearoa New Zealand’s alcohol legislation is that Local Alcohol Policies (LAPs) can be developed by a local authority in consultation with their community to further restrict conditions in their geographical area (Ministry of Justice, 2020), for example, to limit hours alcohol may be sold or restrict the number or density of licensed premises. LAPs have, however, proved to be difficult to implement when faced with opposition from industry and retail interests, as they are subject to appeal and may not be implemented until all appeals have been resolved (Alcohol Healthwatch, 2022). While government legislation bans any form of promotion for tobacco products, advertising of alcohol and fast food are not government regulated. A voluntary code developed by advertisers, advertising agencies and the media calls for advertising to be ‘socially responsible’ (Advertising Standards Authority, undated), but has been widely criticized as ineffective (Sing *et al.*, 2020).

Furthermore, in the policy context of Aotearoa New Zealand, the government has obligations under Te Tiriti o Waitangi. Te Tiriti o Waitangi is an agreement between Māori, the indigenous people of Aotearoa New Zealand and the British Crown in 1840, which facilitated the establishment of government, while acknowledging Māori ‘tino rangatiratanga’—authority in decision making (Orange, 2021). Current alcohol legislation fails to include a reference to Te Tiriti o Waitangi, and this has been criticized by Māori, because of inequitable experiences of harm faced by Māori communities (Maynard, 2022). Existing nutrition policy has failed Māori (Theodore *et al.*, 2015). Furthermore, a lack of government regulation in relation to food marketing has failed to adequately protect Māori children, and therefore could be considered a breach of the Te Tiriti (Theodore *et al.*, 2015; Garton *et al.*, 2022). Changes to the food environment, through the expansion of the digital environment will be considered in line with responsibilities under Te Tiriti from a health equity perspective,

This paper reports on interviews with health policy experts on the health implications of uptake in on-demand delivery of food, alcohol and smoking/vaping products. We discussed their perspectives on the health harms from on-demand delivery and their views on policies that might mitigate these harms. This study aimed to (i) describe policy experts’ views of the health harms from on-demand delivery services, (ii) give expert views on current policies and regulations in relation to health harms and (iii) identify key areas for policy change and improvement. The findings are discussed in the context of international literature.

## METHODS

We used a qualitative research methodology informed by critical theory. Critical theory is relevant to this research topic given the desire to inform policy, reduce inequities and achieve structural change. All authors are public health researchers with a social justice and equity value orientation. Fourteen policy experts were recruited using a purposive sampling strategy. An initial list of potential participants was drawn up based on the authors (R.C., C.M. and S.B.) professional networks and subject expertise. We also recruited through snowballing where these potential participants suggested others. Participants were people that the authors knew of but were not necessarily personally known and were not known by the student interviewers (H.M. and M.C.). Participants were approached directly because they were experts in public policy on alcohol (five participants), food (three), smoking/vaping (three) or in more than one of these areas (three). Participants were employed in a range of central and local government agencies and ministries, health authorities, non-Government Organisations (NGOs) and university research positions in Auckland, Wellington and Christchurch, Aotearoa New Zealand. Although we did not ask participants to report demographic information, three interviewees were Māori Health experts, and others may identify as Māori but were not working specifically in Māori health. We asked participants to describe their experience and all interviewees all had considerable experience and expertise in their field. Interviews took place between October 2021 and June 2022 and were conducted by four authors (H.M.—three interviews, M.C.—six interviews, S.B.—four interviews and C.M.—one interview). Student interviewers were trained by authors with expertise in qualitative research (C.M. and S.B.) and this was iterative as the interviews progressed. Most of the 14 interviews were conducted online for pragmatic and safety reasons during on-going COVID-19 restrictions. All participants were familiar with technology and had reliable connectivity at their workplaces. Participants received an information sheet before their interview and provided written consent. Interviews lasted between 40 min and 1 h. The study was approved by the University of Otago Human Ethics Commitee [No. D21.278].

A semi-structured interview guide (Supplementary Material 1) was used to generate in-depth discussion using a standardized interview structure to ensure consistency across interviewers. The interview guide was developed by authors with topic expertise (C.M. and R.C.) building on previous qualitative research with policy makers (McKerchar *et al.*, 2021). One government department declined to participate. Participants were asked for their perspectives on the current legislative and regulatory frameworks applying to the particular commodity of interest for them, the potential health harms created by on-demand delivery services, and their proposals for policy change to mitigate those harms. Interviewees were free to expand on their concerns as they related to the wider health environment. Interviews were audio-recorded, transcribed verbatim, de-identified and a qualitative descriptive analysis was carried out. Each interview transcript was read carefully several times and coded using an inductive approach [(Bourgeault *et al.*, 2010), p. 469] by one author (S.B.). The codes were then reviewed for common patterns across the full set of data and synthesized into themes in discussion with other authors (C.M. and R.C.). For example, references to advertising that encouraged ‘likes’ on social media were coded as ‘individual targeting’ initially and subsequently merged into the major theme ‘expansion of marketing and promotional methods’. These major themes formed the basis of a descriptive content analysis. Qualitative descriptive studies stay close to the data and do not engage in highly abstract theoretical analysis (Lambert and Lambert, 2012) but rather engage in an ‘exercise of thought, practice of analysis, activity of reflection and interpretation’ that moves beyond description to synthesis and interpretation which may lead to interventions or change [(Bradshaw *et al.*, 2017), p. 6]. This is a relevant and practical approach that aims to gain first-hand knowledge from people about the phenomenon in question (Bradshaw *et al.*, 2017).

To ensure methodological and reporting quality, we have consulted and reported on all items in the Standards for Reporting Qualitative Research (SRQR) checklist (O’Brien *et al.*, 2014).

## RESULTS

The analysis showed that participants’ views of the health harms from on-demand delivery services encompassed three broad themes related to concerns over: (i) the expansion of access to and availability of unhealthy commodities; (ii) the inadequacy of existing restrictions and regulations for the digital environment and (iii) the expansion of personalized marketing and promotional platforms for unhealthy commodities. We have labelled these three broad themes as ‘concerns’ and discuss each in turn below. Participant quotes are used to substantiate the preceding analytic findings. We indicate our health policy experts’ areas of expertise in the excerpts below as follows: alcohol (A), food (F), smoking/vaping (S) and multiple (M).

The three ‘concerns’ were also the starting point for participants’ proposals for policy change to mitigate these health harms. These proposals are presented under the theme of ‘Health policy experts’ proposals to mitigate harms’.

### Concern 1: Increased access and availability of unhealthy commodities

All interviewees were concerned about the dramatic increase in on-demand delivery of unhealthy commodities that had been accelerated by COVID-19 pandemic restrictions:

…as soon as you increase access and availability, you increase that pathway of effects, you increase sales, therefore, consumption…So it’s a huge public health issue … health outcomes, such as mental health, and dental health, so there’s lots of different health outcomes of a public health nature that we would be worried about. (F)

Experts noted that the demand from consumers was a strong economic incentive for more businesses to list their products through on-demand services. The increased access and availability of products then encouraged further uptake by more consumers and businesses. Classifying alcohol as an ‘essential product’ during the lockdown was of particular concern to interviewees, as many retailers, obtained an extension to their licence to allow them to do home delivery (Casswell, 2020; Huckle *et al.*, 2020):

Of course, we did need home based deliveries of some essential foods to people who couldn’t get out, … COVID sort of opened the door and made it more obvious, and people got used to having all this stuff delivered. …alcohol and tobacco and various other products, Coca Cola, whatever … No, they’re not essential either in COVID or, indeed in any other time. And so that’s a policy choice. (M)

After lockdowns ended, consumers had learned that ordering online was an easy, convenient and quick way to have products they wanted brought to their door. The immediate access to addictive products was described as undermining efforts at harm minimization and working against people’s attempts to cut down or quit. Deliveries also assisted heavy drinkers to top up their alcohol when they would have been reluctant to drive somewhere to buy more.

Now you can sit down at home, order your alcohol and food and cigarettes, …and vaping, all from the comfort of your living room …. It just defeats the purpose of what we [health promoting organisations] are trying to do. (M)

Another concern was the impact on domestic violence from the increase in heavy drinking at home:

So, we’ve seen increases in domestic violence in certain localities … which is very much attributed to increased alcohol consumption, which is coming from increased online sales, because it’s happening in the home… [families] who are already more vulnerable than other communities. (A)

Food experts suggested that widespread availability and increased use of on-demand delivery services had changed the food environment, for example, increasing the geographic reach of fast-food outlets, so that a location near to a consumer’s home had become less relevant:

… these delivery services increase the reach of food outlets and are disrupting our traditional food environments… traditionally food environment is defined as something that is a kilometre away from where you are but …people are ­ordering eight to ten kilometres away from themselves. (F)

Moreover, the food on offer was dominated by items of low nutritional value, and even those that claimed to be ‘healthy options’ were misleading.

…people click on that healthy option, assuming that the foods there are healthy … You know, just because something had a bit of lettuce added to it did not make it healthy. (F)

Experts believed that the increased availability and uptake of nutritionally poor food meant that the food environment was being degraded, and there was an additional environmental impact from the packaging needed to keep food fresh and uncontaminated but was then discarded.

### Concern 2: Undermining of regulations for the sale and supply of alcohol and smoking/vaping products

The second area of concern for interviewees was that existing regulations that restricted the sale and supply of alcohol and smoking/vaping products were no longer fit for purpose in the changed digital environment. They drew attention to the way that the regulatory frameworks around the sale and supply of alcohol, smoking and vaping products could easily be circumvented by online services; as long as purchasers could pay, they only needed tick a box to say they were over 18 years of age:

I think everyone was sort of basing themselves around the fact that supposedly people are under 18 can’t get a credit card. I think that was sort of more of the default sort of check. But in fact, that’s been shown that under 18s can get credit cards, or they borrow someone else’s … (A)

Experts also noted that ‘controlled purchase’ checks on adherence to regulations for the sale of alcohol, smoking and vaping products at walk-in stores did not apply to online orders. While the seller had the responsibility of ensuring that the purchaser was not breaking the law, businesses frequently contracted delivery out to couriers or ‘gig economy’ drivers who had an incentive not to check:

… the way the [Sale and Supply of Alcohol] Act is structured, if a courier or delivery on behalf of the licensee, were to knowingly deliver to a minor, they can be held responsible for that. If they knowingly deliver to an intoxicated person, they can be held responsible for that. If they just leave the alcohol and go, there’s no accountability for that in the Act. (A)

Several interviewees described testing the system themselves and reported incidents where alcohol had been left unattended on their doorstep or handed to under-age family members. They also pointed out that on-demand deliveries were frustrating the harm minimization intentions of community LAPs as online retailers were free to deliver outside the agreed hours in the plan and from outside the local area:

… you could knock down two, three bottles stores, but you don’t know how much has been delivered from the bottles store down the road … that could be 10, 15 kilometres between them. …we look at the off licenses licensed in that area. But what we don’t know is what’s coming in, through remote sales. (A)

There were similar concerns around the lack of enforcement of regulations for sales of smoking and vaping products to minors. Experts in monitoring of the regulations described the constant complaints they received from parents and school principals about regulations not being enforced for in-person sales, and lamented that online services had exacerbated the situation: ‘*It [online ordering] was probably one further thing – barrier – removed*.’ (S)

### Concern 3: Expansion of marketing and promotional platforms for unhealthy commodities

The third major area of concern was the proliferation of methods for marketing unhealthy commodities that on-demand services enabled:

The moment that they’ve got your data … you’ve handed over all of that information to them [to] uniquely target you with the type of products that you want to buy, promotions coming up, different types of marketing for particular beverages. (A)

Moreover, algorithms that tracked what people were looking at online were able to target people who had never placed an order:

[We] did a media release on that day to say that it [making alcohol an essential product during lockdown] was the wrong decision. And basically, within 24 hours, my Facebook feed was suddenly full of bottle stores saying we now sell online, you know, come to come to our website… (A)

Alcohol experts spoke of the complaints they had received about socially irresponsible promotions during the COVID-19 lockdowns:

… they were doing some really questionable advertising on their Facebook pages and, and using things like the yellow and white COVID Ministry of Health logos and stuff to advertise alcohol … you know, locked down, get plastered kind of stuff, you know, to, to market their products (A)

Food experts reported that fast food franchises were using promotions that consumers were unlikely to recognize as advertising:

… these large fast food franchises actually pay them money to be the most popular items on their apps… it’s like lobbying for space; lobbying for real estate on the app… the end user thinks it is popular because a lot of people are buying from it … things like KFC or McDonalds because they have the money to pay to get on those popular areas whereas a small business outlet does not have that capacity. (F)

They noted that ‘meal deals’ and ‘value bundles’ were designed to generate further consumption of nutritionally poor foods, especially among young adults where price was an important factor:

This is a public health concern, because value added bundles add excessive amounts of deleterious nutrients, saturated fat, added sugar, sodium to already nutritionally poor foods…. There are other studies that have looked at how young people make purchasing decisions for menu items and if there are meal deals because price purchasing is important for them, that they are strong persuaders. (F)

Interviewees also said they believed the existing code of marketing to children and young people (Advertising Standards Authority, undated) was weak and inadequate:

There is …a code of advertising which talks about the fact that you shouldn’t directly target children. … it also only covers marketing directed to children, and we’re really interested in all the marketing children are exposed to. (M)

They drew attention to strategies that compounded overt promotions such as encouragement from vendors for individuals to ‘like’ or ‘share’ on their social media platforms:

But what really, we haven’t been able to catch up with is that viral sharing of personal people’s Tik Tok’s, Facebook shares, likes … you can’t regulate people from liking and sharing something on their own personal pages yet (F)

### Health policy experts’ proposals to mitigate harms

Overall, interviewees believed that the uptake of on-demand access to unhealthy commodities was creating an environment that increased adverse health outcomes for individuals, and for overall population health. The three ‘concerns’ outlined above were also the starting point for the participants’ proposals for policy change to mitigate harms. These proposals are presented below.

#### Limiting access and availability

Experts noted that there were already mechanisms that would limit access and availability for both alcohol and smoking/vaping products if they were strengthened. They emphasized that online alcohol deliveries needed to be brought into line with the same conditions imposed on walk-in sales and to be subject to the conditions of LAPs that limited hours of sale and limits on the number of licenced premises within a particular area. In relation to limiting access and availability of smoking and vaping products, interviewees pointed to the New Zealand Smoke Free Action plan to de-normalize smoking (Ministry of Health, 2021). They suggested implementing the section of the plan which proposed reducing the number of premises permitted to sell smoking and vaping products. They suggested further that home delivery of smoking and vaping products could simply be prohibited.

Health policy experts proposed that proactive approaches involving young people in developing educational packages were more likely to be effective than messages about harm from addictive products. They pointed out that the harms were already well known and highlighted positive alternatives for money that was being spent on addictive substances, for example, championing young people who were role models:

Those are the stories that we need to hear … if we get our rangatahi [youth] … and then getting an All Black [New Zealand National rugby team] or someone like that standing with him and naming him and saying … I support him, that will make more difference than anything else in our communities here. But use the rangatahi… to say, I know that’s here, but I choose not to and I’m glad I had the courage to do that. Imagine a role model with a family behind him saying totally agree with you… That’s the sort of promotion that we need to do now. Because they will get more hits, more shares, more likes. (S)

Restricting access to unhealthy food was acknowledged by participants to be more difficult than with alcohol and smoking/vaping products, which were already subject to regulation. Reducing the density of food outlets around schools, for example, or preventing new outlets in areas that were already over-supplied with access to unhealthy food, was raised as being desirable. However, most experts believed that it would be difficult to implement, and policy change could potentially create a situation where different pieces of central and local government legislation might come into conflict. Density regulation was said to be impractical in some areas, for example where an existing school and shopping mall with many fast-food outlets were close together. Moreover, with on-demand deliveries covering a wide area, the geographical location of food outlets had become less relevant.

The food experts suggested that a more proactive approach would be developing a coherent national strategy on food and nutrition to provide a framework for addressing the increasingly poor food environments and its effect on population health. Such a strategy would:

… have all the different aspects of the food environment whether it is availability, accessibility, cost of food, promotion of food. (F)

They noted that the over-arching nature of the strategy was critical to ensure that implementation could be done smoothly and avoid central and local government mandates being at odds. They emphasized that it required collaboration between all sectors of government to improve the broader food environment rather than focussing on the behaviour of individuals. A range of central and local government groups with interest in equity, health, welfare and business would need to be involved.

They noted that cross-sector collaboration was encouraged by the Public Service Act (New Zealand Government, 2020a; Public Service Commission, 2022), however, one or two key individuals needed to take the lead to drive change:

…it does need a cohesive approach and it needs people to be joined up. …often there needs to be someone stepping up to get that going. And where it’s not definitely in someone’s mandate, it’s easier for it to fall through the gaps. (F)

Within such a strategy, experts suggested initiatives such as working to improve the quality of the food delivered, exploring how to increase access to healthy food for people who found it difficult to shop in-person, and options such as mandating kilojoule labelling or improving the health star rating and applying it to all food sold, including restaurant meals. Other proposals were for better and more consistent messaging about the impact of high-density fast foods and their impact on health, as well as combining messages about the health impact with the environmental impact of the packaging used in meal deliveries.

#### Updating regulations and boosting enforcement

Experts put forward a range of proposals to update the regulations on the sale of alcohol, smoking and vaping products so that they were applicable and enforceable in the digital as well as the built environment. In relation to alcohol, they said it would require explicit changes to the current legislation (New Zealand Parliament, 2022) that included online sales:

… what this is calling for is a major rewrite … it needs to be more explicit around what you can and can’t do. And we would also call for the fact that for a bottle store that holds an off-licence, that they can’t just sell online as well, they need to apply to sell on-demand. And part of that application will show what procedures they have in place and how they’re going to comply with them. (A)

In parallel, they wanted the rules around obtaining an alcohol off-licence to be tightened so that any business selling alcohol online would be required to have a delivery plan with certain criteria. Participants pointed to a system being implemented in some Australian states which required delivery drivers to be trained to identify intoxicated recipients and minors (New South Wales Government, 2021; Victorian Gambling and Casino Control Commission, 2021).

Some participants proposed policies that further restricted online purchases of age-restricted products by requiring individuals to register with each seller they bought from and provide robust age authentication at the time of registration. Delivery would then be available only to registered customers and would prevent both under-age ordering and impetuous ordering by non-registered customers. All interviewees emphasized that new regulations must be supported with adequate funding and an expanded compliance workforce, to make sure they were effectively monitored and enforced.

#### Limiting promotion and marketing

All experts wanted promotion and advertising of unhealthy commodities to be restricted, especially the marketing of ‘junk’ (or discretionary) food to children:

… when it comes to restricting junk food marketing, and the exposure that children see of junk food marketing, we will be calling for a total ban on digital advertising… You know that obviously [they would] still be able to advertise their services but the type of foods that they would be able to promote as being deliverable would have to change. (M)

They were, however, pessimistic about how such a ban might realistically be implemented. Only one expert believed that there was some capacity to act:

… we can’t regulate cross border marketing. … what we’ve found is actually, a lot of these companies have a New Zealand base or a New Zealand presence of a New Zealand Company Entity that therefore brings them under the mandate of New Zealand government to regulate them. (F)

Most interviewees believed that much advertising originated from companies located outside of Aotearoa New Zealand and could not be restricted by the government, especially promotions that worked through pop-ups on people’s social media pages.

Overall, participants were aware that the proposals they put forward would not be easy to implement and that each category of commodity had specific challenges. They noted that a strong evidence base was needed to document the impact of on-demand delivery services on health and equity. Once the evidence was established, experts believed health and community groups needed to work collaboratively on a tightly focussed campaign to build public support for change and resist lobbying from industry interests. They suggested that influential and respected champions from academia and civil society should spearhead campaigns to mobilize public sentiment.

Getting the public’s mind around the idea that [the current situation] is detrimental to the public health and not nanny state is going to be a hard sell as well. (F)

They noted that proposed policy changes needed to be specific, with a focus on the mechanism by which they might be achieved, and clarity on who was being regulated—the provider, or the online delivery services. Enforcement and technical issues would need to be thought through and the penalties for breaching any regulations specified.

## DISCUSSION

Health policy experts highlighted three main areas of concern about the potential health harms as more consumers have shifted to online purchases of unhealthy food, alcohol and smoking/vaping products. Their concerns encompassed the increase in access and availability exacerbated by COVID-19 restrictions; the inadequacy of existing regulation for the digital environment and the proliferation of personalized marketing and promotions. These themes echoed the concerns expressed in many international studies about the way that online purchasing has undermined attempts to promote health that were not designed for the current virtual/digital environment (Montgomery *et al.*, 2019; Tatlow-Golden and Garde, 2020; Jankhotkaew *et al.*, 2022; Ling *et al.*, 2022). To address the harms related to on-demand delivery, health policy experts proposed strategies to tighten restrictions and use proactive policy approaches to encourage healthier food options. Their suggestions were largely consistent with proposals that have been made across the international literature.

Internationally, approaches to reducing access and availability of unhealthy food have largely focussed proactively on creating an environment to encourage consumers to make healthier food choices. In our study, the development of a comprehensive national food strategy (currently absent in Aotearoa New Zealand), was proposed as a strategy to mitigate health harms. Such a strategy would then work to improve the quality of food sold through the on-demand delivery services, mandate labelling of all foods and promote consistent messages about a healthy diet. This approach to on-demand delivery is also being recommended in Europe [e.g. (De Schutter *et al.*, 2020; Halloran *et al.*, 2022)], to ensure that existing regulations are adapted and applied to the digital food environment, that popular food items are reformulated to reduce the sugar, saturated fat and salt content, and that there are clear and consistent public health messages. A report from the WHO (WHO, 2021) highlighted the potential of on-demand delivery services to promote healthier meal choices, citing a systematic review (Wyse *et al.*, 2021) where this approach had a positive impact on purchases of healthier options. Findings from Australia also highlight the potential of on-demand delivery services to promote healthier options and ‘nudge’ consumers towards such options (Bates *et al.*, 2020). However, given the limited healthy food options currently available in Aotearoa New Zealand, and the buy-in required by on-demand delivery services, this is somewhat optimistic.

Proposals for alcohol largely relate to strengthening and enforcing existing restrictions by extending them to on-demand services for rapid delivery (Mojica-Perez *et al.*, 2019; Casswell, 2020; Noyes *et al.*, 2021; Fitzgerald *et al.*, 2022). They include tightening procedures for online age authentication and requiring specific training for delivery drivers following the example of similar changes that have been introduced in parts of Australia [e.g. (Fitzgerald *et al.*, 2022)]. In July 2021, the New South Wales Government (Australia) introduced new regulations that made it an offence to deliver alcohol to a minor or an intoxicated person, tightened hours for delivery and required that persons delivering alcohol had passed a training course so that they were acting according to the new regulations (New South Wales Government, 2021; Australasian College for Emergency Medicine, 2022). From June 2022, same day delivery providers were also required to have systems in place to authenticate the identity and age of new and existing customers. The Australian state of Victoria has tightened the law in a similar way, instituting new requirements for licensees and delivery drivers as well as penalties for not verifying age and intoxication status on delivery (Victorian Gambling and Casino Control Commission, 2021). Since our interviews were conducted, there has been substantial change to planned alcohol policy reform in Aotearoa New Zealand. The New Zealand government made the decision not to support changes to alcohol policy that seemed likely to stop the lengthy appeals by the alcohol industry bodies and retailers that have blocked community wishes for LAPs. It remains unclear whether any future alcohol reform will address the issue of online deliveries from outside LAPs.

Restricting access to on-demand delivery of smoking and vaping products does not appear to have received the same level of attention that has been given to on-demand delivery of alcohol, either in Aotearoa New Zealand studies or internationally. While The Smokefree 2025 goals in Aotearoa New Zealand are moving towards reducing the number of outlets that are permitted to sell tobacco products (Ministry of Health, 2021), they do not specifically refer to on-demand delivery. Similarly, efforts to restrict youth vaping both in Aotearoa New Zealand and internationally, are more concerned with overall uptake regardless of how and where vaping products are accessed (O’Connor *et al.*, 2019; Ball *et al.*, 2021; Reynolds and Winickoff, 2021; Burrows, 2022). Since completing the interviews for our study, there has been a recent increase in checks on vape stores and fines imposed where the law is being disregarded (Radio New Zealand, 2022), indicating that there is more emphasis on enforcing the current law, even if not focussed on the digital space. As mentioned in our study, proactive efforts have also been promoted that encourage young people towards healthier lifestyles and away from addictive products. These include incentives to engage in sports, mentoring by sports coaches, participating in community action and the involvement of young people themselves in creating educational packages that discourage vaping (Popova *et al.*, 2021; Reynolds and Winickoff, 2021; Royal New Zealand College of General Practitioners, 2022). Future research should investigate the effectiveness of proactive efforts to encourage young people away from addictive products, thereby informing future policy efforts.

Proposals and recommendations for these policy changes, however, are not necessarily straight forward to implement in a constantly changing digital environment (Jankhotkaew *et al.*, 2022), where industry players exploit loopholes where regulations and policy are unclear (Colbert *et al.*, 2021; Noyes *et al.*, 2021; Ling *et al.*, 2022). Barriers include resource constraints, legal loopholes, low priority among decision-makers, limited capacity for implementing further regulation, and gaps in knowledge that would assist decision making (Noyes *et al.*, 2021). It also takes time for policies to be established and regulations to be defined, implemented and enforced, and the time lag has allowed marketers to ‘reinvent’ products so that they can stay in business and force regulators to constantly ‘play catch up’ [(Ling *et al.*, 2022), p. 225].

Policy change that could restrict marketing and promotion of unhealthy commodities has been even harder to achieve. The digital environment for unhealthy commodities is underpinned by ‘big data’ that uses artificial intelligence and machine learning coupled with enhanced connectivity from the exponential growth in digital devices and smartphone use to target consumers personally (World Health Organisation, 2021). The online services collect large amounts of data, building customer profiles and targeting individuals based on their personal preferences and previous purchases (Casswell *et al.*, 2021; World Health Organisation, 2021). They expose the inadequacy of regulations designed for the built environment where consumers shopped in-person and advertising was disseminated through billboards and television (Carah and Brodmerkel, 2021; Kelly *et al.*, 2021; Scobie *et al.*, 2022). Māori concerns about data sovereignty in e-commerce are currently inadequately protected in trade agreements (Waitangi Tribunal, 2023). Further research is needed to explore data sovereignty in relation to on-demand delivery to adequately protect Māori data sovereignty. Future research could also explore the extent of marketing strategies used to target consumers and identify where regulation is most needed.

Nevertheless, there are some examples where action has been taken. The State of Victoria in Australia has introduced restrictions on alcohol advertising that targets minors, promotes violence or is otherwise ‘not in the public interest’ (Victorian Gambling and Casino Control Commission, 2021). Several countries in Europe have implemented restrictions on alcohol advertising across public space, print, broadcast and social media (Casswell *et al.*, 2021; Scobie *et al.*, 2022). Lithuania prohibits all online alcohol advertising, while Finland was the first country to pass legislation specifically directed at social media intended to be shared by consumers (Scobie *et al.*, 2022). These restrictions, however, are recognized to have limitations. Meacham *et al.* found that Apple’s removal of vaping-related apps from the App Store had minimal effect (Meacham *et al.*, 2020). A Finnish report (Kauppila *et al.*, 2019) found that alcohol producers were becoming more skilful at using social media platforms for marketing purposes and that the restrictions had not affected their ability to engage the public (Casswell *et al.*, 2021; Scobie *et al.*, 2022). Moreover, users readily access social media platforms that are hosted outside of these countries so that in-country restrictions do not apply (Katainen *et al.*, 2020). To enforce restrictions would require access to the source codes and algorithms that are highly secretive between ‘Big Alcohol’ and ‘Big Tech’ (Room and O’Brien, 2021). While this appears unlikely at present, there are current challenges to the power of Amazon, Apple, Facebook and Google over legal but harmful content (UK) and in relation to their use of personal information they collect (Australia) (Room and O’Brien, 2021).

Rather than impose restrictions which can be readily undermined, further research and greater knowledge of the digital ecosystem and online behaviour is needed so that evidence to drive policy change is strengthened (Jankhotkaew *et al.*, 2022; Ling *et al.*, 2022). The CLICK framework developed by the WHO European Office (Bica *et al.*, 2020; Tatlow-Golden and Garde, 2020) for example, focusses on understanding the digital ecosystem, documenting how it is operating, and sharing knowledge in order to restrict marketing that breaches the tenets of the United Nations Committee on the Rights of the Child (United Nations Committee on the Rights of the Child, 2021). Monitoring digital systems also enables regulators to keep pace with new marketing methods (Casswell *et al.*, 2021; Ling *et al.*, 2022) that extend beyond paid advertising to paying for content in posts generated through online groups and influencers (Kelly *et al.*, 2021) and the manipulation of consumers to engage in marketing strategies themselves. Furthermore, it is important to consider the context that consumer behaviour and proposed/enacted regulations are occurring in. In Aotearoa New Zealand, this means exploring behaviours and regulations/policy in relation to our obligations under Te Tiriti o Waitangi.

Better understanding the digital environment opens up the potential not only to take restrictive measures but also to use data analytics and digital technologies to harness food apps so that they become a force for improving diets and thereby reducing obesity and chronic disease (Montgomery *et al.*, 2019; World Health Organisation, 2021). To be successful, widespread collaboration is needed within countries and across borders (Casswell *et al.*, 2021; Tatlow-Golden *et al.*, 2021; World Health Organisation, 2021; Jankhotkaew *et al.*, 2022). Public health advocates, consumer and community groups, civil rights organizations, researchers and research organizations need to work together to develop coherent strategies (Louie *et al.*, 2022), advocate for stronger health policies and greater industry responsibility. Casswell *et al.*, for example, describe a series of ‘next steps’ for promoting alcohol control policies using a multi-stakeholder collaboration across disciplines to build the evidence base, focus research on evaluating the impact of policy options and engage in knowledge translation to disseminate and increase the uptake of research findings [(Casswell *et al.*, 2021), pp. 26–27]. To enable strategies and health policies to remain impactful, they must be supported with adequate funding, an expanded compliance workforce to ensure effective monitoring and enforcement, and clearly specified penalties for breaching regulations.

Governments can be urged to learn from what has worked in other countries, develop ‘coherent approaches across unhealthy commodities… [and] impose positive obligations to target marketing away from specific groups’ [(Casswell *et al.*, 2021), p. 27]. Evidence can be accumulated by inter-country collaboration and used to reduce the power of ‘big data’ and prevent the undermining of efforts that support the health of populations (Montgomery *et al.*, 2019; Tatlow-Golden and Garde, 2020). The ultimate aim might be to develop an international code of marketing for particular commodities such as that which already exists for the promotion of breast milk substitutes (WHO, 2017).

### Strengths and limitations

The strengths of our study include the range of health policy experts from different institutions across the public and non-government sector who were interviewed. Talking with these experts allowed us to explore on-demand delivery in relation to Aotearoa New Zealand regulations across three different categories of unhealthy commodities. The high degree of consistency between the proposals they put forward for policy change and those that are being progressed worldwide emphasizes the urgent international need to address health-related harms from on-demand delivery services. The study also has a number of limitations. On-demand delivery is not a static phenomenon but continually evolves as companies find new ways to increase their customer base and evade regulations that rein them in (Ling *et al.*, 2022). Inevitably, our discussions with health policy experts were limited by the current state of knowledge that prompted our questions, and the participants’ experiences that underpinned their responses at the time. Additionally, our discussions focussed on health harms and did not directly address wider social implications such as the poor work conditions for delivery drivers or those who work in businesses that provide the products (Goods *et al.*, 2019; Poelman *et al.*, 2020). A key example in this respect is the existence of ‘dark’ or ‘ghost’ kitchens (Bates *et al.*, 2020; Rinaldi *et al.*, 2022; Shapiro, 2022), where food is prepared solely for delivery by teams of workers doing a single task under intense time pressure and digital surveillance. Nor did we explore in depth the environmental impact of the travel miles for delivery drivers or the extra packaging that is needed to keep food clean and safe but is then discarded into landfill (Li *et al.*, 2020). While not an immediate health impact, the interdependence of human health and environmental health is well documented (Willett *et al.*, 2019).

## CONCLUSION

The proliferation of on-demand delivery of unhealthy food, alcohol and smoking/vaping products presents a range of potential public health harms through the digital environment. Health policy experts in Aotearoa New Zealand highlighted harms related to increased access and availability of unhealthy commodities, exacerbated by the COVID-19 restrictions; the inadequacy of existing restrictions and regulations to control access in a digital environment; and the expansion of personalized marketing and promotional platforms which can undermine the personal efforts to reduce consumption of unhealthy products. Our health policy experts offered comprehensive solutions to address harms across these key areas. The solutions involved the urgent need to update existing regulations appropriate to the digital sale and supply of alcohol and vaping products, while also emphasising the necessity of cross-sector collaboration to strengthen knowledge of public health harms and build lasting support for change. Collectively, these findings can inform future research and public health policy decisions to address harms and promote better individual and public health.

## Supplementary Material

daad091_suppl_Supplementary_Material
