# Supplementary material for: Promoting health in the digital environment: health policy experts’ responses to on-demand delivery in Aotearoa New Zealand
Source: Health Promot Int. 2023 Aug 23;38(4):daad091. doi: 10.1093/heapro/daad091 (PMC10446141; doi:10.1093/heapro/daad091)
Supplement: daad091_suppl_Supplementary_Material [file daad091_suppl_supplementary_material.docx]

**Introduction**

Thank you for agreeing to speak with me today about the regulation of app-based on demand access to unhealthy commodities. For the purposes of this study, unhealthy commodities are considered to represent unhealthy food and beverages, alcohol, and tobacco products.

**Interview questions**

**Current role and responsibilities**

Can you please tell me about your job?

How is your job related to access to unhealthy commodities?

Can you tell me about your level of knowledge or experience concerning regulation of access to unhealthy commodities?

**Perspectives on app-based on-demand services**

Before we contacted you about this project, had you thought much about app-based on-demand delivery services?

Did/do you consider these services to be an emerging issue of concern?

**Public health implications of these services**

Do you see any public health implications of increasing access to unhealthy commodities by these services?

What about health equity implications?

**Policy and regulation related to app-based on-demand services**

How do you think these services are being regulated now? This may differ for alcohol, food and nicotine products.

From your professional role, what policies exist that are relevant to these services? Has any policy been developed specifically for these services?

Are you aware of any compliance activities currently undertaken in relation to these services? If so, can you please describe these?

Do these services present any challenges to policy and regulation of access to unhealthy commodities?

Do you see any issues in how regulation of these services might occur?

Is any new regulation or policy might be required in relation to these services?

At what level of government do you think regulation of on-demand app-based services should occur?

Do you see any barriers in relation to regulating these services?

**Closing**

Finally, we would like to interview other people who can provide insight in this area. Is there anyone else you suggest we interview about this?

Is there anything else you would like to add?

Would you like a copy of the research findings?

Thank you again for your valuable insight into this area and for offering you time to help with this research project. I am very grateful for your help.
